# Atomically precise copper dopants in metal clusters boost up stability, fluorescence, and photocatalytic activity

**DOI:** 10.1038/s42004-023-00817-5

**Published:** 2023-02-08

**Authors:** Yifei Zhang, Jingjing Zhang, Zhiwen Li, Zhaoxian Qin, Sachil Sharma, Gao Li

**Affiliations:** 1 Institute of Catalysis for Energy and Environment, College of Chemistry and Chemical Engineering Shenyang Normal University, Shenyang, 110034 China; 2 State Key Laboratory of Catalysis, Dalian Institute of Chemical Physics, Chinese Academy of Sciences, Dalian, 116023 China; 3 University of Chinese Academy of Sciences, Beijing, 100049 China; 4 School of Advanced Sciences, Department of Chemistry, Vellore Institute of Technology, Andhra Pradesh (VIT-AP university), Amaravati, Andhra Pradesh 522237 India

**Keywords:** Nanoparticles, Nanoparticle synthesis, Optical materials, Photocatalysis

## Abstract

The structurally precise alloy nanoclusters have been emerged as a burgeoning nanomaterial for their unique physical/chemical features. We here report a rod-like nanocluster [Au_12_Cu_13_(PPh_3_)_10_I_7_](SbF_6_)_2_ (Au_12_Cu_13_), which was generated through a transformation of a [Au_9_(PPh_3_)_8_]^3+^ intermediate in the presence of CuI, unveiled by time-dependent UV-vis spectroscopy, electrospray ionization mass spectrometry as well as single crystal X-ray diffraction. Au_12_Cu_13_ is comprised of two pentagonal bipyramids Au_6_Cu units and a pentagonal prism Cu_11_ unit, where the copper and gold species are presented in +1 and 0 chemical states. The Cu-dopants significantly improved the stability and fluorescence (quantum yield: ~34%, 34-folds of homo-Au_25_(PPh_3_)_10_Br_7_). The high stability of Au_12_Cu_13_ is attributed to the high binding energy of iodine ligands, Au-Cu synergistic effects and its 16-electon system as an 8-electron superatom dimer. Finally, the robust Au_12_Cu_13_ exhibited high catalytic activity (~92% conversion and ~84% methyl formate-selectivity) and good durability in methanol photo-oxidation.

## Abstract (web summary)

Copper doping of atomically precise gold nanoclusters is a useful strategy to tune their chemical and physical properties, but Au–Cu nanocluster alloys tend to exhibit poor stability. Here, a [Au_12_Cu_13_(Ph_3_P)_10_I_7_](SbF_6_)_2_ cluster is prepared and shown to display enhanced stability and fluorescence in comparison to homonuclear cluster [Au_25_(PPh_3_)_10_Br_7_](SbF_6_)_2_, in addition to promising photocatalytic activity for methanol oxidation.

## Introduction

In the famous lecture in 1959 with the title “*There is plenty of room at the bottom*”, R. Feynman predicted the beginning of the research at the atomic level^1^. His prediction has been come true in a real sense with the emergence of research in the area of magic nanoclusters, where we can make nanoscale things at the atomic level in a highly controlled fashion^2^. Such functionalized nanoscale materials at the atomic level actually gained significant importance in technology progression due to their tunable physical and chemical properties^3,4^. Serving as an important part of such nanomaterials, the metal nanoclusters “M_*n*_L_*m*_”, where *n* and *m* represent the number of metal atoms and surficial protecting ligands (L), respectively, have been highly promising in both fundamental research and practical applications, such as sensing^5^, photo-luminescence^6^, catalysis^4,7,8^ and bio-imaging^9,10^, owing to their unique and modulating physicochemical properties.

Metal atom doping is an important strategy to tune the structure and properties of metal nanoclusters^11–15^. Recently, the copper species doped into gold nanoclusters to generate Au-Cu alloy composites can well tailor their electronic structures and in turn to boosting the intrinsically physical and chemical properties, especially in luminescence and catalysis^13,16–21^. For example, Jin et al. have prepared an Au@Cu alloy nanocluster with a 71.4% quantity yield (QY) of fluorescence^18^. We found that the copper atoms doped in M_25_(SC_2_H_4_Ph)_18_ clusters, primarily enhanced the benzaldehyde-selectivity in the oxidation of styrene^20^. Unfortunately, Au-Cu alloy nanoclusters tend to show less stable under external environments^21–23^ (e.g., light irradiation, oxidizer, and thermal conditions) due to the nature of Cu^I^ and Cu^0^ species, corroborated by the basis of Jellium Model^12^. Therefore, copper clusters and Au-Cu alloy clusters were often acquired under extreme conditions and stored in cool and dark place filled with inert atmosphere. The poor stability of Cu-based clusters becomes a burning issue and is still a big challenge for their applications.

Focused on the tunable properties and the improvement of stability of Cu doped gold clusters, we, herein, designed a strategy to prepare the rod-like [Au_12_Cu_13_(Ph_3_P)_10_I_7_](SbF_6_)_2_ (abbreviated as Au_12_Cu_13_, hereafter) alloy cluster based on the self-assemble behavior of [Au_9_(PPh_3_)_8_](SbF_6_)_3_ (Au_9_ in short) clusters in the presence of CuI, which was further monitored by a serious of time-dependent measures including ultraviolet-visible (UV-vis) spectroscopy, electrospray ionization mass spectrometry (ESI-MS), and single crystal X-ray diffraction (SCXRD) to conclude the assembling process/mechanism of Au_12_Cu_13_ nanocluster. To our surprised, although Au_12_Cu_13_ cluster shares similar structure with the rod-like [Au_13_Ag_12_(PPh_3_)_10_Cl_8_](SbF_6_)^14^ and [Au_25_(PPh_3_)_10_Br_7_](SbF_6_)_2_ (noted as Au_25_ hereafter) clusters obtained in similar way, the dopant of Cu atoms in this alloy cluster has boosted its stability and photoluminescence significantly. The synergistic effects of Au and Cu atoms and strong Cu-I bonds may be the reason for the strong and high fluorescence of Au_12_Cu_13_ clusters. Finally, the Au_12_Cu_13_ cluster-based photo-catalyst was prepared based on the high stability and unique photo-properties, which shown excellent catalytic activity in the selective photo-oxidation of methanol.

## Results

### Synthesis and structure determination

**Fig. 1:**
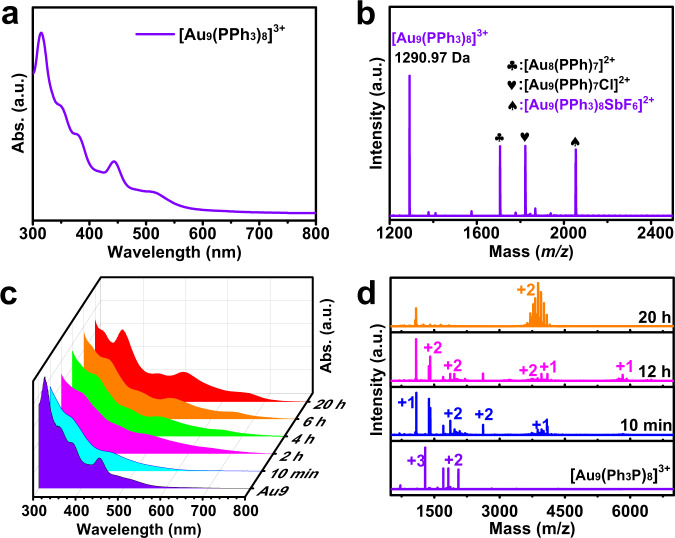
The conversion of Au_9_ to Au_12_Cu_13_ clusters. **a** UV-vis spectrum (in CH_2_Cl_2_) and **b** positive ion mode ESI-MS of Au_9_ cluster. Conversion of Au_9_ to Au_12_Cu_13_ clusters monitored by **c** time-dependent UV-vis spectra and **d** time-dependent ESI-MS.

The alloy nanoclusters with composition of [Au_12_Cu_13_(Ph_3_P)_10_I_7_](SbF_6_)_2_ were prepared in a step-by-step synthetic strategy. Briefly, a clear solution obtained upon mixing Ph_3_PAuCl and AgSbF_6_ was reduced by NaBH_4_ giving a dark-brown mixture, which was further reacted with CuI to produce the target clusters. To figure out the progress of alloy cluster formation, we firstly monitored the reaction solution by UV-vis spectroscopy and ESI-MS before the CuI addition. The obtained red-brown mixture gave five obvious peaks at 315, 350, 378, 443, and 511 nm in UV-vis spectrum (Fig. 1a), similar to that of the well-known [Au_9_(PPh_3_)_8_]^3+^ clusters^24^. And four main mass peaks at 1290.45 Da, 1705.90 Da, 1822.5 Da, and 2054.49 Da in the scale of *m/z* 200–10,000 Da, assigned to the compositions of [Au_9_(PPh_3_)_8_]^3+^ (*m/z* (*z* = 3) 1290.33 Da calcd.), [Au_8_(PPh_3_)_7_]^2+^ (*m/z* (*z* = 2) 1705.85 Da calcd.), [Au_9_(PPh_3_)_7_Cl]^2+^ (*m/z* (*z* = 2) 1822.3 Da calcd.), and [Au_9_(PPh_3_)_8_SbF_6_]^2+^ (*m/z* (*z* = 2) 2054.37 Da calcd.), respectively, were detected in positive ion mode of ESI-MS (Fig. 1b and Supplementary Figure 1), which confirmed the existence of [Au_9_(PPh_3_)_8_]^3+^ clusters in the obtained mixture. Note that such fragmentations are usual owing to the dissociation during ESI ionization of these phosphine protected metal nanoclusters^14^. All these results suggested that Au_9_ cluster is dominant and served as starting material/precursor for the further construction of Au_12_Cu_13_ cluster.

Next time-dependent UV-vis spectroscopy (TD-UV-vis) and ESI-MS were applied to monitor the construction process of Au_12_Cu_13_ cluster originated from the reaction of Au_9_ cluster and CuI in solution. As presented in Fig. 1c, the intensity of characteristic absorption peaks of Au_9_ cluster at 315 and 443 nm decreased within initial 10 min, accompanied with the color deepening after CuI addition. Further, the peaks at 352, 434, 508, and 655 nm increased gradually in TD-UV-vis, indicating that the Au_9_ cluster was transforming into other clusters. Figure 1d showed that the mass signals corresponding to Au_9_ cluster disappeared quickly, and a series of new peaks (Supplementary Table 1) were shown up, demonstrating that Au_9_ cluster reacted with CuI rapidly generating some metastable clusters, such as [AuCu(Ph_3_P)_2_I]^+^, [Au_3_(Ph_3_P)_3_CuI]^+^, [Au_6_(Ph_3_P)_6_]^+^, [Au_8_Cu(Ph_3_P)_8_I]^2+^ and [Au_9_Cu(PPh_3_)_8_I_2_]^2+^. These metastable clusters were further aggregated with each other to furnish Au_25-x_Cu_x_ clusters during the time-consuming thermodynamic process. Similar to the silver-doped Ag_x_Au_25−x_ nanoclusters^15^, the number of Cu dopants in M_25_ cluster ranges from 1 to 9, which is lower than that of the final Cu atoms (13) in the tested crystal sample. It may indicate that the Cu-doping process keeps going on during the crystallization process and the Au_12_Cu_13_ should be the most robust one in the final products^14^.

**Fig. 2:**
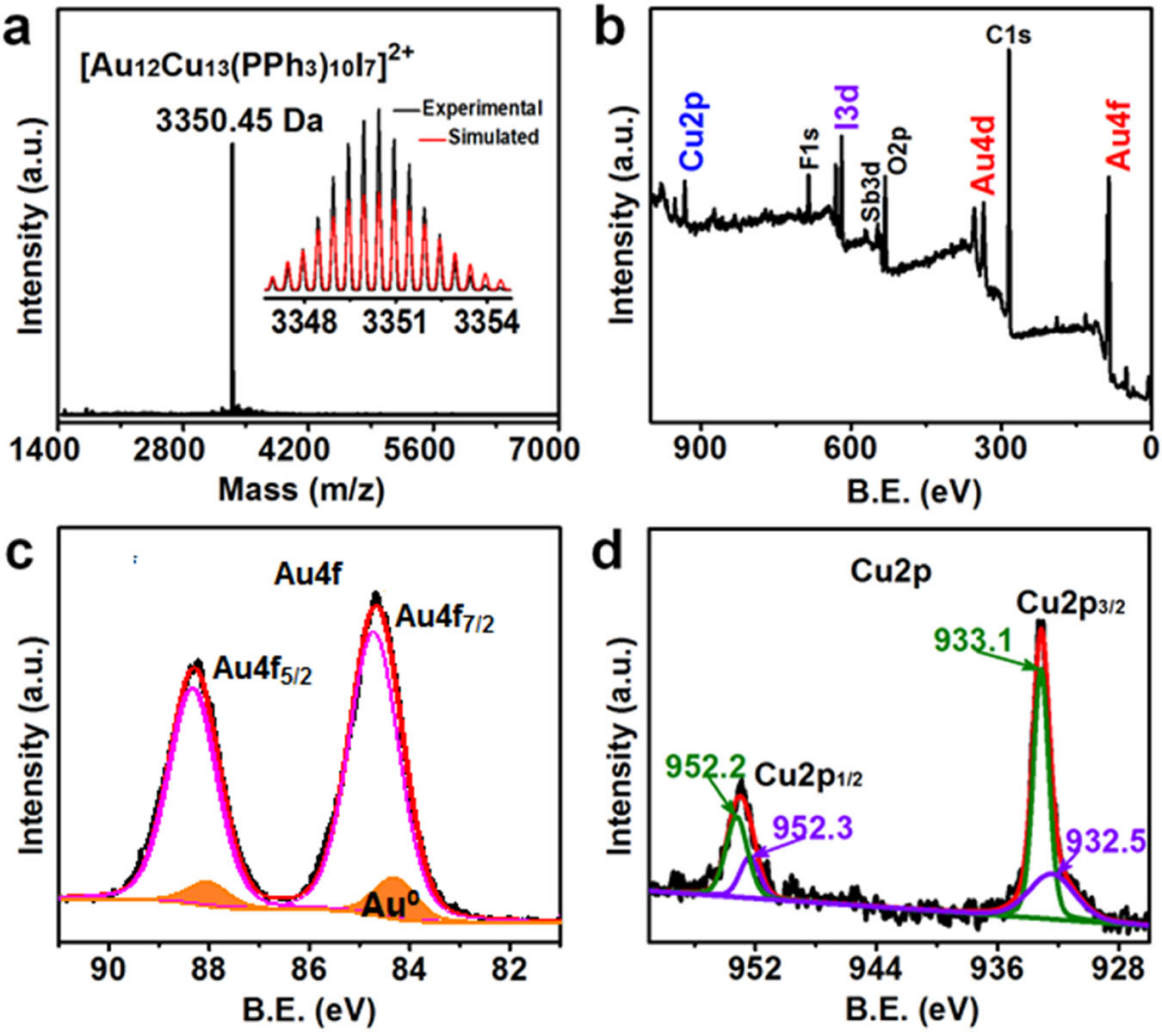
Physical property of the Au_12_Cu_13_ nanocluster. **a** ESI-MS with isotopic pattern (insert). **b** Wide scan XPS. **c** Au 4 f and **d** Cu 2p XPS spectra of Au_12_Cu_13_ nanocluster.

The composition and purity of the obtained Au_12_Cu_13_ crystals were manifested by ESI-MS and X-ray photoelectron spectroscopy (XPS). As shown in Fig. 2a, the ESI-MS spectrum exhibited an intense peak at *m/z* ~3350.45 Da (z = 2, calcd. *m/z* ~3350.44 Da for Au_12_Cu_13_P_10_C_180_H_150_I_7_, deviation: 0.01 Da) in the scale from *m/z* ~1400 to 7000, corresponding to [Au_12_Cu_13_(PPh_3_)_10_I_7_]^2+^ cluster with high molecular purity. The isotopic pattern was found to be in exact agreement with simulated one and the peaks separation of *m/z* ~ 0.5 Da, confirming the +2 charged state of Au_12_Cu_13_ nanocluster. These results demonstrated that the cluster specie in crystals is pure [Au_12_Cu_13_(PPh_3_)_10_I_7_]^2+^ instead of Au/Cu alternation and the Au_12_Cu_13_ cluster exhibit a 16-electron system (i.e. 25 (metal atoms) – 7 (I atoms) – 2 (charge) = 16).

The bimetal nature of Au_12_Cu_13_ cluster was also characterized by the wide scan XPS, Fig. 2b. The binding energy (BE) of Au 4f_7/2_ in Au_12_Cu_13_ can be deconvoluted to Au^0^ (highlighted in orange) and Au^I^ species^25^ (Fig. 2c). Taking note of previous investigations, the final-state hole-shielding effect arising from extra atomic relaxation acts in opposite to electron donating effect of phosphine ligands, resulting in the increase of binding energy^26–28^. An unsymmetrical BE peak of Cu 2p_3/2_ near 933.1 eV was presented in Cu 2p XPS (Fig. 2d), excluding the existence of Cu^II^ species (934.2 eV for Cu 2p in Cu^II^)^29^. And the Cu 2p_3/2_ peak also can be deconvoluted into two peaks, corresponding to Cu^0^ species at 932.5 eV and Cu^I^ at 933.1 eV^30^. For validation, Auger electron spectrum showed a broad peak at 571.0 eV with a 1.0 eV positive shift with respect to the value of Cu^I^ (570.0 eV) and a weak peak to Cu^0^ at 568.0 eV (Supplementary Figure 2), indicating the presence of both Cu^+^ and Cu^0^ species in Au_12_Cu_13_. And the Cu^0^ species, generally, should be attributed to the shared vertex Cu atom, while the Cu^I^ one should belong to the peripheral and end vertex Cu atoms bonded directly with I atoms according to SCXRD analysis (*vide infra*). To the best of our knowledge, the Au_12_Cu_13_ cluster in present work is the first case of AuCu alloy cluster, where the Cu species exists both in +1 and 0 of chemical states, without any partial occupancy.

Based on the results of ESI-MS and UV-vis, we put forward explanation that the Au_12_Cu_13_ cluster should be prepared via the transformation of Au_9_ clusters upon a reaction with CuI. Thus, we conceived a sequence of experiments for the transformation of Au_9_ clusters into another M_25_ clusters, including rod-like Au_25-y_Ag_y_(PPh_3_)_10_Cl_7_ (Au_25-y_Ag_y_) and homo Au_25_(PPh_3_)_10_Br_7_ (Au_25_) in the presence of AgCl and KBr, respectively. Further, with respect to the self-assembly of these clusters, their structural analyses are of the essence. Therefore, the crystals of Au_12_Cu_13_ and Au_25_ were grown by slow vapor diffusion of diethyl ether into a CH_2_Cl_2_ solution of clusters at ~10 °C over two weeks and characterized by SCXRD. Of note, the Au_25-y_Ag_y_ nanoclusters have been extensively studied^14,31^.

**Fig. 3:**
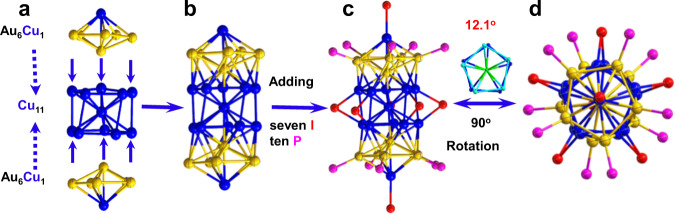
Configuration of Au_12_Cu_13_ cluster. **a**, **b** Anatomy of Au_12_Cu_13_ cluster. **c** Side view and **d** top view. Color code: Au, yellow; Cu, blue and cyan; I, red; P, pink. Note that the other atoms are omitted for clarity.

SCXRD analysis revealed that Au_12_Cu_13_ and Au_25_ clusters crystallize in *P21/n* and *P21/m* space groups, respectively. They share similar rod-like framework, as illustrated in Supplementary Figure 3. The metal core of Au_12_Cu_13_ can be divided into one Cu_11_ and two Au_6_Cu_1_ units (Fig. 3a). Two Au_6_Cu_1_ units bind with the Cu_11_ unit through Au-Cu metal bonds, generating an Au_12_Cu_13_ framework (Fig. 3b). The obtained Au_12_Cu_13_ metal kernel/core is directly ligated by ten PPh_3_ ligands through Au-P bonds and seven I^-^ ions via Cu-I bonds furnishing the full structure of Au_12_Cu_13_ cluster. In detail, five I anions are coordinated to ten Cu atoms in Cu_11_ unit taking a *µ*_2_-bridging coordination mode (i.e., one I anion per two Cu atoms of Cu_11_ unit with an average Cu-I bond length of ~2.586(2) Å), and the remaining two I anions are bonded to the vertex Cu atoms, Fig. 3c. In another way, the rod-like Au_12_Cu_13_ cluster could be deemed as the fusion of two icosahedron Au_6_Cu_7_ units by sharing a Cu vertex like the well-known rod-like M_25_ (M = Au, Ag, Cu) clusters^14,15,32^. Of note, Au_12_Cu_13_ cluster is different from Cu_x_Au_25–x_(PPh_3_)_10_(PhC_2_H_4_S)_5_Cl_2_ with an eclipsed arrangement in structure, where the copper atoms partially occupy the top and M_11_ sites and bonded with chlorine or thiolate ligands^33^.

In a typical way, the core structure of Au_12_Cu_13_ and Au_25_ can be regarded as a four-layer cylinder^32^, Supplementary Figure 4. It is worth noting that the two neighboring pentagons of layer II and layer III in Au_12_Cu_13_ showed a staggered arrangement and adopt a torsion angle of 12.1^o^ (Fig. 3d). In comparison, no twisting is observed in Au_25_ cluster (Supplementary Figure 4b), indicating that the Cu atoms cause the torsional stress in Au_12_Cu_13_ cluster, which is plausibly attributed to the different atomic radii of Au (~144 pm) and Cu (~128 pm). Both Au_12_Cu_13_ and Au_25_ kernels are protected by triphenylphosphine and halide (I/Br) ligands in similar coordination patterns. The iodide anions and phosphine ligands coordinate selectively to Cu and Au atoms in Au_12_Cu_13_ cluster, respectively, due to the different electro negativities of Cu and Au.

Furthermore, the various M-M, M-P and M-X (M = Au/Cu, and X = I^-^/Br^-^) bond distances are summarized in Supplementary Table 2. The Au_c_-Au_p_ and Au_p_-P bond lengths (c and p denote central and peripheral) of Au_12_Cu_13_ and Au_25_ are found comparable. And the average Cu_c_-Cu_p_ (2.717(2) Å) and Cu_p_-Cu_p_ (2.761(2) Å) bond distances in Cu_11_ unit of Au_12_Cu_13_ were found to be substantially smaller than the corresponding Au_c_-Au_p_ (2.878(2) Å) and Au_p_-Au_p_ (2.940(3) Å) in Au_25_ cluster. Interestingly, compared with these structures of Au_12_Cu_13_, Au_25_ and Au_13_Ag_12_ clusters, they all can deemed as two vertex-sharing icosahedrons with a metal center, which further promote us to associate them with the uncomplete-icosahedral Au_9_ cluster with an Au center, the starting materials as well. In short, the Cu atoms in system not only lead the reconstruction and assemble of Au_9_ clusters into Au_12_Cu_13_ clusters, but also caused the structural distortion in the Au_12_Cu_13_ clusters.

### Stability of Au_12_Cu_13_ clusters

**Fig. 4:**
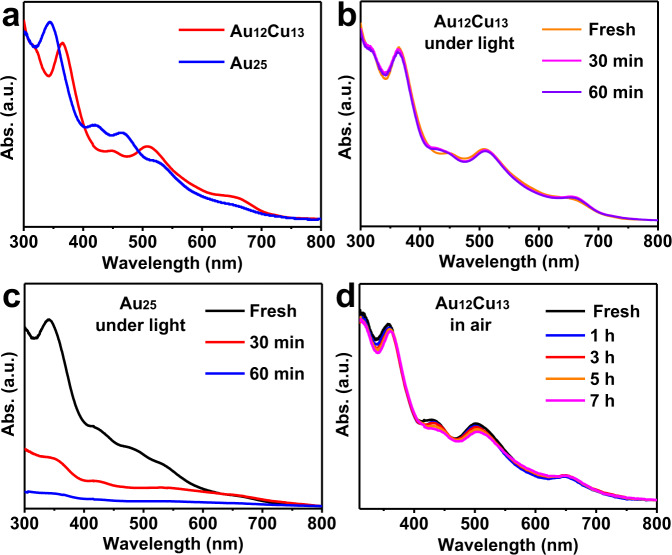
Stability of Au_12_Cu_13_ clusters. **a** UV-vis spectra of Au_12_Cu_13_ and Au_25_ nanoclusters. Stability tests of Au_12_Cu_13_ and Au_25_ clusters: **b**, **c** under sunlight and **d** in air.

The optimum stability of metal nanocluster is essential for their use in various applications, and it is previously reported that the Cu dopants in gold nanoclusters often caused the instability to the alloy clusters^22,34^. Therefore, we examined the stability of Au_12_Cu_13_ *vis-á-vis* that of Au_25_ clusters in solution. As shown in Fig. 4a, Au_12_Cu_13_ in a CH_2_Cl_2_ solution give three obvious absorption features at 448, 508 and 655 nm in the range of 400 to 800 nm, which is notably much distinct from those of Au_25_ (419, 470, 526 and ~660 nm), indicating reasonable perturbation of electronic structure upon Cu doping.

The stability of Au_12_Cu_13_ and Au_25_ clusters in CH_2_Cl_2_ was evaluated under irradiation of sunlight via time-dependent UV-vis spectroscopy using crystal samples. As depicted in Fig. 4c, UV-vis profile of homo-Au_25_ decreased obviously in 30 min and almost disappeared after 60 min indicating the poor stability of homo-Au_25_ cluster in CH_2_Cl_2_. Whereas the profile Fig. 4b assigned to Au_12_Cu_13_ clusters kept intact illustrating Au_12_Cu_13_ cluster is higher stability than corresponding homo-Au_25_ clusters owing to the dopant of Cu atoms. Furthermore, the stability of Cu species in Au_12_Cu_13_ cluster have been improved significantly in solution exposed to air since these UV-Vis profiles in Fig. 4d remained overlapping over 7 hours and no precipitation formed, which is unusual even for Cu^I^ complexes. Hence the Au_12_Cu_13_ with high stability in solution under light or in air is achieved, which is not observed in other copper doped M_25_ nanoclusters^22^. Although the rod-shaped clusters of Au_12_Cu_13_ and Au_25_ share the similar configuration and a 16-electron system (i.e. the dimer of 8-electron superatom)^35^, the composition in core differs suggesting the synergistic effects between Au and Cu may be an important reason for the high stability of Au_12_Cu_13_ cluster. Secondly, the ligated I^-^ ions with large radius (γ: ~ 2.2 Å) at waist of Au_12_Cu_13_ cluster exhibit higher coordinate capability than Br^-^ (γ: ~1.96 Å) in Au_25_ cluster.

### Fluorescence property

**Fig. 5:**
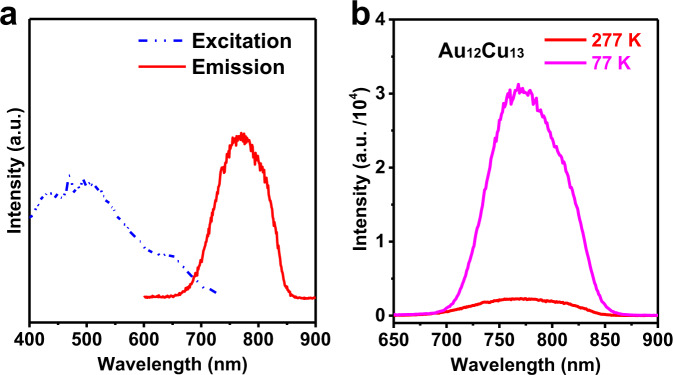
Fluorescence property of Au_12_Cu_13_ clusters. **a** Excitation and emission spectra of Au_12_Cu_13_ cluster in solution. **b** Temperature-dependent fluorescence of Au_12_Cu_13_ clusters in solution.

We next investigated the fluorescence (FL) property of Au_12_Cu_13_ nanocluster. The excitation curve from 400 nm to 700 nm in Fig. 5 shares the similar profile with UV-vis spectrum of Au_12_Cu_13_ monitored by 774 nm, suggesting the fluorescence of Au_12_Cu_13_ generated from the metal core cluster and different from small metal-organic complexes with similar ligands, whose fluorescence need to be pumped by UV lower than 400 nm. Interestingly, the intensity of fluorescence has been enhanced significantly once the Cu atoms were doped. An obvious broad fluorescence emission centered at 774 nm was noticed in the wavelength range from 660 to 850 nm, excited at λ_ex_ ~ 470 nm as depicted in Fig. 5, which is broader than that of Au_25_ clusters (700–850 nm, Supplementary Figure 5). Further, the maximum emission wavelength of Au_12_Cu_13_ at λ_em_ ~ 774 nm was found red shifted with respect to Au_25_ clusters (λ_em_ ~ 750 nm, λ_ex_ ~ 470 nm) and Au_12_Ag_13_ (λ_em_ ~ 739 nm, λ_ex_ ~ 430 nm) clusters^14^. Besides, the excitation spectrum monitored at 774 nm emission (Fig. 5a, blue dash line) was found almost identical to the absorption spectrum of Au_12_Cu_13_ cluster (Fig. 5a, red line), and Stokes shift of Au_12_Cu_13_ cluster was determined as 304 nm close to that of Ag atoms doped Au_12_Ag_13_ cluster (309 nm)^14^ and smaller than that of Au_25_ cluster (330 nm). The quantum yield (QY) of Au_12_Cu_13_ was determined to be ~34% by an absolute method, which is significantly higher than Au_12_Ag_13_ (QY: ~26%)^14^ and Au_25_ clusters (~1%, by absolute method) and close to the highly fluorescent [Au_12_Ag_13_(PPh_3_)_10_(SR)_5_Cl_2_]^2+^ nanocluster (~40%)^15^, where the 13 Ag was doped in the center position similar to our Cu-centered Au_12_Cu_13_ cluster.

Furthermore, the FL lifetime of Au_12_Cu_13_ is up to 900 ns deduced from Supplementary Figure 6, and an almost linear fading tendency was detected indicating the nature of monoexponential FL decay dynamics of Au_12_Cu_13_ cluster in solution. These results illustrated the synergistic effect of Cu and Au atoms in Au_12_Cu_13_ clusters plays a key role in the drastic enhancement of fluorescence. We further investigated the temperature-dependent fluoresce (TDFL) of Au_12_Cu_13_ clusters at 277 and 77 K, Fig. 5b. An obvious enhancement of about 10-fold was achieved when the cluster solution was cooled to 77 K from 277 K, and FL emission kept unchanged, suggesting no-radiative transmittance Au_12_Cu_13_ at low temperature decreased obviously. TDFL analysis proves that FL of Au_12_Cu_13_ may be originated from the same excited state and the electron structure of Au_12_Cu_13_ is not temperature dependent^18^.

### Catalytic test in photo-oxidation of methanol

**Fig. 6:**
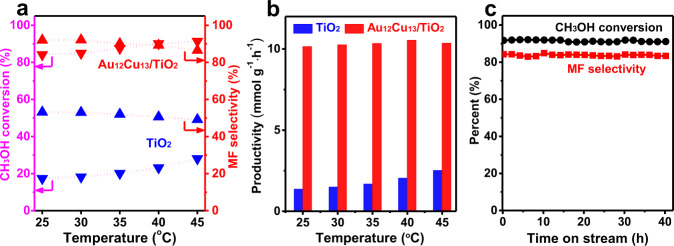
Catalytic performance of Au_12_Cu_13_/TiO_2_ in the photo-oxidation of methanol. **a** Catalytic performance as a function of temperature on TiO_2_ (P25) and Au_12_Cu_13_/TiO_2_. **b** Formation rate of methyl formate (MF) over TiO_2_ and Au_12_Cu_13_/TiO_2_ catalysts. **c** Durability test of the Au_12_Cu_13_/TiO_2_ catalysts at 25 °C. Reaction conditions: ~ 20 mg catalysts, λ = 365 nm, methanol (1.0 v %) and O_2_ (0.5 v %) balanced with N_2_ at the flow rate of 20 mL min^−1^.

The structurally well-identified and highly stable Au_12_Cu_13_ clusters with characteristic absorption features and excellent fluorescence can serve as a promising photocatalyst towards oxidation of methanol to methyl formate as both gold and copper species are considered as excellent candidates for this transformation^14,29,36,37^. Thus, we tested Au_12_Cu_13_ clusters as catalyst for photo-oxidation of methanol. The catalytic experiment details were given in Experimental section. In brief, ~0.5 wt% of Au_12_Cu_13_ nanocluster was firstly loaded on surface of TiO_2_, followed by treatment of Au_12_Cu_13_/TiO_2_ samples with amorphous Al_2_O_3_ using atomic layer deposition (ALD) technique to grow the shell/cage of Al_2_O_3_ (100 ALD cycles) around the Au_12_Cu_13_ cluster at 150 °C^38^, where Au_12_Cu_13_ clusters keep inert. The photocatalytic results are depicted in Fig. 6 and Supplementary Figure 7. It is worthy to note that no methanol conversion was detected when the light and catalysts were absent, demonstrating that the photo-oxidation occurred over the Au_12_Cu_13_/TiO_2_ catalysts. And it is found that methanol conversion was gradually improved, and methyl formate-selectivity was gradually decreased with the increasing reaction temperatures (from 25 to 45 °C), as shown in Fig. 6a. The highest methyl formate formation rate over Au_12_Cu_13_/TiO_2_ is evaluated to be ~10.6 mmol g^−1^ h^−1^ at 40 °C, which is 4-folds of that over TiO_2_ (Fig. 6b) and substantially higher than previously reported photocatalysts, Supplementary Table 3^39^.

Further, the durability of Au_12_Cu_13_/TiO_2_ catalyst for the photooxidation of methanol was tested. Significantly, during the whole process (~40 h), the cluster catalyst gave rise to a constant ~92% methanol conversion and a ~84% methyl formate-selectivity with no appreciable loss of activity, as shown in Fig. 6c. These results strongly indicate the robust nature of the Au_12_Cu_13_ composite, holding promise of outstanding catalytic activity for the prolonged period of time.

We have developed a strategy to acquire the rod-like [Au_12_Cu_13_(PPh_3_)_10_I_7_](SbF_6_)_2_ nanocluster, which can also be extended to the preparation of [Au_25_(PPh_3_)_10_Br_7_](SbF_6_)_2_ and [Au_25-y_Ag_y_(PPh_3_)_10_CI_8_](SbF_6_) nanoclusters. A plausible mechanism for the Au_12_Cu_13_ formation that Au_12_Cu_13_ clusters were constructed by the dimerization of metastable intermediates like [Au_8_Cu(Ph_3_P)_8_]^2+^ and [Au_9_Cu(Ph_3_P)_8_I]^2+^generated from the reaction of Au_9_ clusters and CuI was presented and demonstrated by UV-vis spectroscopy, ESI-MS and single crystal X-ray diffraction technologies. The obtained Au_12_Cu_13_ cluster exhibits high stability in solution and outstanding photo-luminescence character with QY ≈ 34%, which laid the foundation for the applications in photoluminescence and photo-catalysis. Furthermore, the Au_12_Cu_13_ clusters showed good catalytic performance in the photo-oxidation of methanol toward methyl formate and good durability. The present work deepens the understanding of the assembling mechanism of clusters and provide the future guidelines for the highly controllable synthesis of functionalized alloy nanoclusters.

## Methods

### Synthesis of M_25_ clusters

Typically, Ph_3_PAuCl (25 mg) reacted with AgSbF_6_ (17.2 mg) in 4 mL of CH_2_CL_2_ and methanol (v/v = 1), and the obtained clear solution was reduced by NaBH_4_ solution (2 mg dissolved in ice-cold methanol) at 0 ^o^C, giving a dark brown solution. After ~24 h of stirring, CuI (9.5 mg) was added into the mixture and kept stirring at 0 °C. The solution was filtrated when the characteristic peaks at 352, 434, 508 and 655 nm appeared. The filtrate was further evaporated to dryness under vacuum, which was further washed twice with a mixture of hexane and dichloromethane (v/v = 5:1). The pure clusters were finally extracted with a mixed solution of CH_2_Cl_2_ and CH_3_OH (v/v = 1). A black block crystal of Au_12_Cu_13_ was obtained via a slow diffusion of diethyl ether into the dichloromethane solution of clusters over two weeks. Yield: 15.25 mg, 50.5% (based on Au). The Au_25_ (yield: ~58%) and Au_25-x_Ag_x_ (yield: ~49%) clusters were obtained through a similar method by using KBr (11 mg, 0.05 mmol) and AgCl (7 mg, 0.05 mmol) to replace CuI, respectively. The detailed characterizations of the clusters are given in in the SI.

### Catalytic performance evaluation

The Au_12_Cu_13_/TiO_2_ catalyst was prepared by the method of atomic layer deposition; see details in the SI. The oxidations of methanol to methyl formate were carried out in a home-made continuous-flow aluminum alloy reactor with a rectangle quartz window on the top. A 500 W high-pressure mercury lamp (CEl-LAM 500) with a wavelength of 365 nm was employed as the light source, installed above the quartz window of the reactor with the light intensity of 18.6 mW cm^−2^. The reaction temperatures were controlled at 25–40 °C using a cooling water circulation. 20 mg of catalysts were uniformly coated on the glass’s surface, which is fully exposed to light irradiation. A gas mixture containing 1.0 v% methanol, 0.5 v% O_2_ balanced with N_2_ at the flow rate of 20 mL·min^−1^ was introduced into reactor that is bubbled through a liquid methanol in a flask. The effluent was examined by an on-line Agilent 7820 with a thermal conductivity detector (TCD) and a flame ionization detector (FID).

### Further details

See Supplementary Methods for details on X-ray crystallographic structural determinations, additional nanocluster characterizations, and preparation of the Au_12_Cu_13_/TiO_2_ catalyst.

## Supplementary information

Peer Review File

Supplementary Information

Description of Additional Supplementary Files

Supplementary Data 1

Supplementary Data 2

## Data Availability

Crystallographic data for [Au_12_Cu_13_(Ph_3_P)_10_I_7_](SbF_6_)_2_ and [Au_25_(Ph_3_P)_10_Br_7_](SbF_6_)_2_ is deposited in The Cambridge Crystallographic Data Centre (CCDC) as CCDC-1965910 and CCDC-1966985 (Supplementary Data 1 and 2). The data supporting the findings of this study are available within this article and its Supplementary Information. Extra data are available from the corresponding author upon reasonable request.

## References

[1] Feynman, R. P. There’s Plenty of Room at the Bottom an Invitation to Enter a New Field of Physics (1960).

[2] Chakraborty I, Pradeep T (2017). Atomically Precise Clusters of Noble Metals: Emerging Link between Atoms and Nanoparticles. Chem. Rev..

[3] Kawawaki T (2020). Controlled colloidal metal nanoparticles and nanoclusters: recent applications as cocatalysts for improving photocatalytic water-splitting activity. J. Mater. Chem. A.

[4] Jin R, Li G, Sharma S, Li Y, Du X (2021). Toward Active-Site Tailoring in Heterogeneous Catalysis by Atomically Precise Metal Nanoclusters with Crystallographic Structures. Chem. Rev..

[5] Chen LY, Wang CW, Yuan Z, Chang HT (2015). Fluorescent Gold Nanoclusters: Recent Advances in Sensing and Imaging. Anal. Chem..

[6] Kang X, Zhu M (2019). Tailoring the Photoluminescence of Atomically Precise Nanoclusters. Chem. Soc. Rev..

[7] Shi Q, Qin Z, Sharma S, Li G (2021). Recent Progress in Heterogeneous Catalysis by Atomically and Structurally Precise Metal Nanoclusters. Chem. Rec..

[8] Qin Z (2020). A Homoleptic Alkynyl-Ligated [Au_13_Ag_16_L_24_]^3-^ Cluster as a Catalytically Active Eight-Electron Superatom. Angew. Chem. Inter. Ed..

[9] Porret E, Le Guevel X, Coll JL (2020). Gold Nanoclusters for Biomedical Applications: Toward in Vivo Studies. J. Mater. Chem. B.

[10] Polavarapu L, Manna M, Xu QH (2011). Biocompatible Glutathione Capped Gold Clusters as One- and Two-Photon Excitation Fluorescence Contrast Agents for Live Cells Imaging. Nanoscale.

[11] Kang X, Li Y, Zhu M, Jin R (2020). Atomically precise alloy nanoclusters: syntheses, structures, and properties. Chem. Soc. Rev..

[12] Takano S, Tsukuda T (2021). Chemically Modified Gold/Silver Superatoms as Artificial Elements at Nanoscale: Design Principles and Synthesis Challenges. J. Am. Chem. Soc..

[13] Hakkinen H (2008). Atomic and Electronic Structure of Gold Clusters: Understanding Flakes, Cages and Superatoms from Simple Concepts. Chem. Soc. Rev..

[14] Qin Z (2022). Tailoring Optical and Photocatalytic Properties by Single-Ag-Atom Exchange in Au_13_Ag_12_(PPh_3_)_10_Cl_8_ Nanoclusters. Nano Res.

[15] Wang S (2014). A 200-Fold Quantum Yield Boost in the Photoluminescence of Silver-Doped Ag_x_Au_25-x_ Nanoclusters: the 13th Silver Atom Matters. Angew. Chem. Int. Ed..

[16] Koshevoy IO (2008). Self-assembly of supramolecular luminescent Au(I)-Cu(I) complexes: “wrapping” an Au_6_Cu_6_ cluster in a [Au_3_(diphosphine)_3_]^3+^ “belt”. Angew. Chem. Int. Ed..

[17] Yang H (2013). Ligand-stabilized Au_13_Cu_x_ (x = 2, 4, 8) bimetallic nanoclusters: ligand engineering to control the exposure of metal sites. J. Am. Chem. Soc..

[18] Song Y (2021). Ultrabright Au@Cu_14_ Nanoclusters: 71.3% PhosphoRescence Quantum Yield in Non-degassed Solution at Room Temperature. Sci. Adv..

[19] Jia JH, Liang JX, Lei Z, Cao Z-X, Wang QM (2011). A luminescent gold(I)-copper(I) cluster with unprecedented carbon-centered trigonal prismatic hexagold. Chem. Commun..

[20] Li G, Jin R (2016). Atomic level tuning of the catalytic properties: Doping effects of 25-atom bimetallic nanoclusters on styrene oxidation. Catal. Today.

[21] Song Y (2020). Atomically resolved Au_52_Cu_72_(SR)_55_ nanoalloy reveals Marks decahedron truncation and Penrose tiling surface. Nat. Commun..

[22] Gottlieb E, Qian H, Jin R (2013). Atomic-Level Alloying and De-Alloying in Doped Gold Nanoparticles. Chem. Eur. J..

[23] Yang H (2014). Structural evolution of atomically precise thiolated bimetallic [Au_12+n_Cu_32_(SR)_30+n_]^4-^ (n = 0, 2, 4, 6) nanoclusters. J. Am. Chem. Soc..

[24] Wen, F., Englert, U., Gutrath, B. & Simon, U. Crystal structure, electrochemical and optical properties of [Au_9_(PPh_3_)_8_](NO_3_)_3_. *Eur. J. Inorg. Chem*. 106–111 (2008).

[25] Zheng K (2017). Motif Mediated Au_25_(SPh)_5_(PPh_3_)_10_X_2_ Nanorod of Conjugated Electron Delocalization. Nano Res..

[26] AbdulHalim LG (2015). Ag_29_(BDT)_12_(TPP)_4_: A Tetravalent Nanocluster. J. Am. Chem. Soc..

[27] Silva DN (2010). A Bioinspired Approach for Controlling Accessibility in Calix[4]Arene-Bound Metal Cluster Catalysts. Nat. Chem..

[28] Menard LD (2006). Sub-Nanometer Au Monolayer-Protected Clusters Exhibiting Molecule-Like Electronic Behavior: Quantitative High-Angle Annular Dark-Field Scanning Transmission Electron Microscopy and Electrochemical Characterization of Clusters with Precise Atomic Stoichiometry. J. Phys. Chem. B.

[29] Shi Q (2019). CuO/TiO_2_ Heterojunction Composites: An Efficient Photocatalyst for Selective Oxidation of Methanol to Methyl Formate. J. Mater. Chem. A.

[30] Platzman I, Brener R, Haick H, Tannenbaum R (2008). Oxidation of Polycrystalline Copper Thin Films at Ambient Conditions. J. Phys. Chem. C..

[31] Qin Z (2020). Atomically Precise Nanoclusters with Reversible Isomeric Transformation for Rotary Nanomotors. Nat. Commun..

[32] Kang X, Zhu M (2019). Intra-cluster growth meets inter-cluster assembly: The molecular and supramolecular chemistry of atomically precise nanoclusters. Coord. Chem. Rev..

[33] Yang S (2017). Crystal Structures of Two New Gold-Copper Bimetallic Nanoclusters: Cu_x_Au_25–x_(PPh_3_)_10_(PhC_2_H_4_S)_5_Cl_2_^2+^ and Cu_3_Au_34_(PPh_3_)_13_(^*t*^BuPhCH_2_S)_6_S_2_^3+^. Inorg. Chem..

[34] Negishi Y, Munakata K, Ohgake W, Nobusada K (2012). Effect of Copper Doping on Electronic Structure, Geometric Structure, and Stability of Thiolate-Protected Au_25_ Nanoclusters. J. Phys. Chem. Lett..

[35] Qin Z (2021). Photo-Induced Cluster-to-Cluster Transformation of [Au_37-x_Ag_x_(PPh_3_)_13_Cl_10_]^3+^ into [Au_25-y_Ag_y_(PPh_3_)_10_Cl_8_]^+^: Fragmentation of A Trimer of 8-Electron Superatoms by Light. J. Phys. Chem. Lett..

[36] Shi Q (2020). Experimental and mechanistic understanding of photo-oxidation of methanol catalyzed by CuO/TiO_2_-spindle nanocomposite: Oxygen vacancy engineering. Nano. Res..

[37] Hou S, Huang M-H, Xiao F-X (2022). Stabilizing atomically precise metal nanoclusters as simultaneous charge relay mediators and photosensitizers. J. Mater. Chem. A.

[38] Zhang S (2022). Surface Isolation of Single Metal Complexes or Clusters by a Coating Sieving Layer via Atomic Layer Deposition. Cell Rep. Phys. Sci..

[39] Shi Q, Wei X, Raza A, Li G (2021). Recent Advances in Aerobic Photo-Oxidation of Methanol to Valuable Chemicals. ChemCatChem.

