# Supplementary material for: Atomically precise copper dopants in metal clusters boost up stability, fluorescence, and photocatalytic activity
Source: Commun Chem. 2023 Feb 8;6:24. doi: 10.1038/s42004-023-00817-5 (PMC9908894; doi:10.1038/s42004-023-00817-5)
Supplement: Supplementary file 2 — Supplementary Information [file 42004_2023_817_MOESM2_ESM.pdf]

Supplementary Information

Atomically Precise Copper Dopants in Metal Clusters Boost up Stability, Fluorescence, and Photocatalytic Activity

Yifei Zhang,[1,2#] Jingjing Zhang,[2,3,#] Zhiwen Li,[2] Zhaoxian Qin,[2,3,*] Sachil Sharma,[2] and Gao Li[2,3,*]

[1] Institute of Catalysis for Energy and Environment, College of Chemistry and Chemical Engineering Shenyang Normal University, Shenyang 110034, China.

[2] State Key Laboratory of Catalysis, Dalian Institute of Chemical Physics, Chinese Academy of Sciences, Dalian 116023, China

[3] University of Chinese Academy of Sciences, Beijing 100049, China.

Supplementary Methods

X-ray Crystallographic Structural Determinations

The Reflection data was collected on a Xcalibur, Atlas, Gemini ultra diffractometer. The crystal was kept at 127 K during data collection. Data reduction, cell refinement and experimental absorption correction were performed with the software package of CrysAlis^{Pro} 1.171.39.38a (Rigaku Oxford Diffraction, 2017). The crystal structure was solved by intrinsic phasing methods using SHELXT 2015[1] and refined by full-matrix least-squares against F^2 using SHELXL 2015[2]. All non-hydrogen atoms were refined anisotropically. Hydrogen atoms were generated geometrically. All calculations were carried out by the program package of Olex2 (*ver*. 1.2.10)[3]. We focused on the clusters structure and counterions, without finding the solvent molecular from weak diffraction spots. Some residual electron cloud (4.5) near metal atoms were caused by the Fourier truncation ripples.

Crystallographic data for $[Au_{12}Cu_{13}(Ph_3P)_{10}I_7](SbF_6)_2$: monoclinic, $P2_1/n$, a = 25.910(5) Å, b = 29.552(6) Å, c = 26.240(5) Å, β = 100.04(3)°, V = 19784(7) Å^3, Z = 4, T = 100 K, 27924 reflections measured, R1 = 0.0668 and wR2 = 0.1791. CCDC-1965910. Crystallographic data for $[Au_{25}(Ph_3P)_{10}Br_7](SbF_6)_2$: monoclinic, $P2_1/m$, a = 16.5758(10) Å, b = 23.3811(16) Å, c = 29.8017(14) Å, β = 103.085(5)°, V = 11250.1(12) Å^3, Z = 2, T = 100 K, 42912 reflections measured, R1 = 0.0405 and wR2 = 0.0973. CCDC-1966985.

Characterization

UV-vis spectra were measured on Shimadzu UV-1800 spectrophotometer. The mass spectra were performed on an ion trap mass spectrometer (Thermofisher LTQ). Positive mode was chosen for the experiments (capillary voltage 33 V). Fluorescence, QY, and lifetime were recorded on a QM 400 spectrofluorometer (PTI). XPS measurements were performed under ultrahigh vacuum (UHV, 1.0×10^{-7} Torr), an axis HS mono chromatized Al K_{α} cathode source of 150 W, a focused X-ray 100 μm beam, a pass energy of 55 eV with 0.1 eV step length, and a detect angle (take off) of 45° on an X-ray microprobe (ULVAC-PHI Quantera SXM). The binding energy was calibrated with that of C1s (284.6 eV).

Preparation of $Au_{12}Cu_{13}/TiO_2$ Catalyst

Typically, 5 mg of $Au_{12}Cu_{13}$ cluster was dissolved in CH_2Cl_2 followed by the addition of TiO_2 powder (1 g) under rapid stirring. When the color of solution faded, the solid was isolated from the mixture by centrifugation (10000 rpm, 2 min), which was then washed twice with CH_2Cl_2 and dried under vacuum as the atomic layer deposition (ALD) precursor. The solid was dispersed into ethanol and then sprayed on the surface of a glass board forming a sample film. The glass board with sample was further installed in ALD system with a trimethylaluminum source and water at 150 ^{o}C for 100 ALD cycles to form $Au_{12}Cu_{13}/TiO_2$ catalyst. Of note, TiO_2 used as reference was treated at the same time.

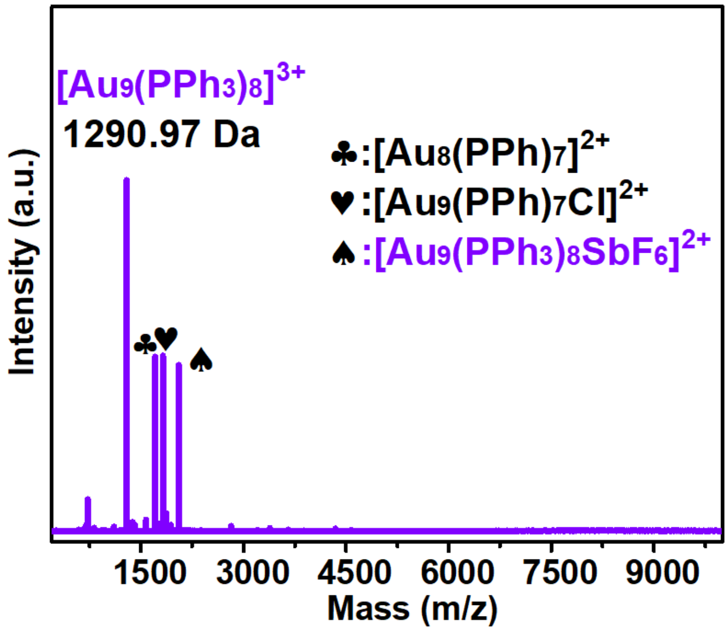

Supplementary Figure 1: Species in reaction mixture detected by ESI-MS before the addition of CuI in the scale of 200 to 10000 Da in positive model.

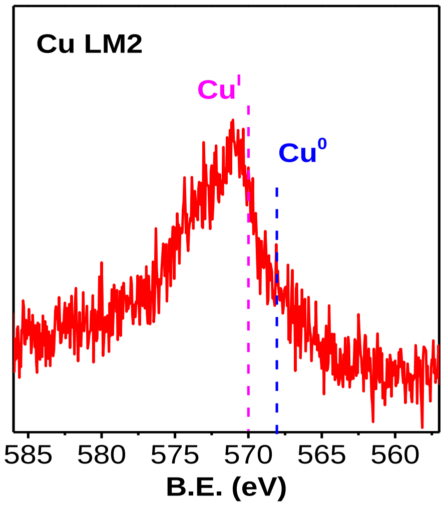

Supplementary Figure 2: Auger electron spectra of Cu species in $Au_{12}Cu_{13}$ cluster.

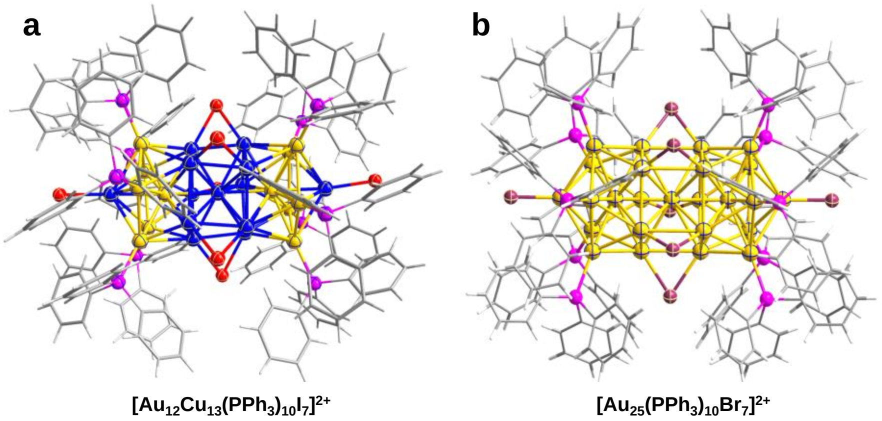

Supplementary Figure 3: Full crystal structures of (a) $Au_{12}Cu_{13}$ and (b) Au_{25} nanoclusters. Color codes: Au, yellow; Cu, blue; I, red; Br, brown; P, pink; Sb, green; F, cyan; C, grey; H, white.

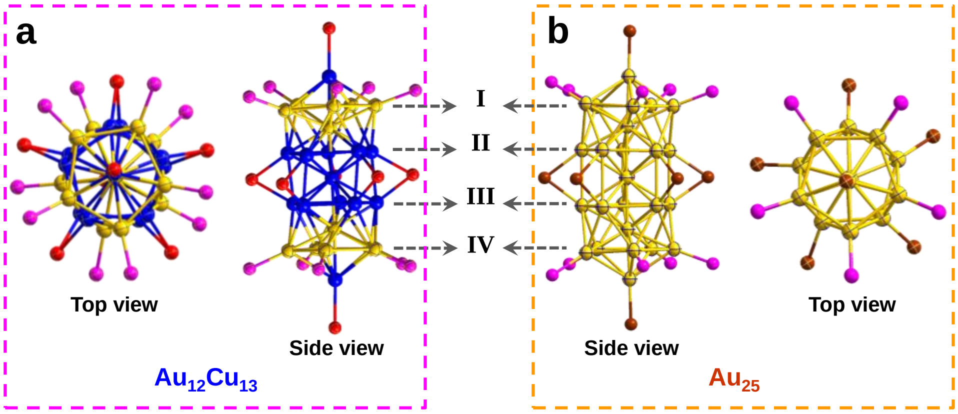

Supplementary Figure 4: Top views of (a) $Au_{12}Cu_{13}$ and (b) Au_{25} with their respective side views in staggered-eclipsed-staggered arrangement of I-IV pentagons. Color code: Au, yellow; Cu, blue; I, red; Br, brown; P, pink; other moieties are omitted for clarity.

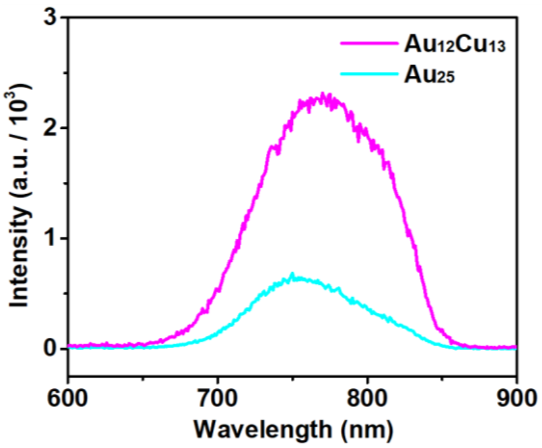

Supplementary Figure 5: The comparison on FL spectra of $Au_{12}Cu_{13}$ (the purple curve) and Au_{25} (the light blue curve) clusters.

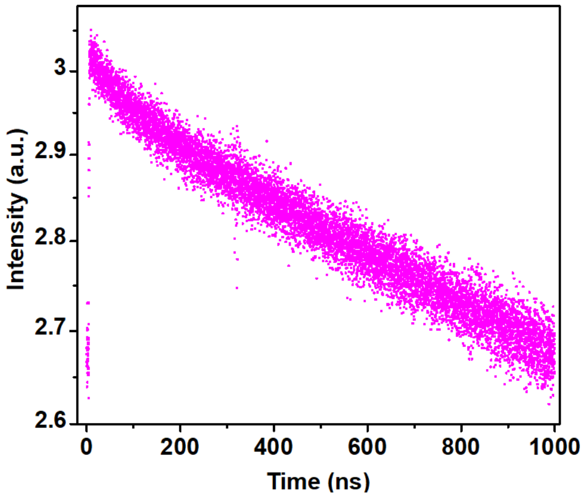

Supplementary Figure 6: The FL decay dynamics of $Au_{12}Cu_{13}$ clusters.

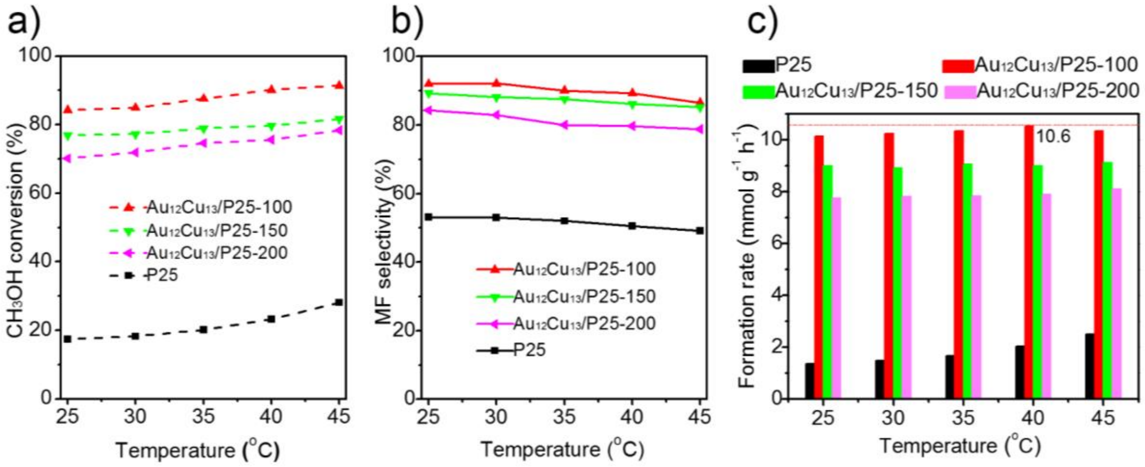

Supplementary Figure 7: Catalytic performance in the photo-oxidation of methanol. Catalytic performance as a function of temperature over $Au_{12}Cu_{13}$/P25-x (x presents the ALD cycle numbers): **a** methanol conversion, **b** selectivity toward methyl formate (MF), and **c** MF formation rate. Reaction conditions: ~ 20 mg catalysts, $\lambda = 365$ nm, methanol (1.0 v %) and O_2 (0.5 v %) balanced with N_2 at the flow rate of 20 mL min^{-1}. Note that the catalytic performance of $Au_{12}Cu_{13}$/P25-x decreased with the increasing ALD cycle numbers, thus the ALD cycle number of 100 was optimal.

Supplementary Table 1: The species detected in time-dependent ESI-MS.

Entry	Species	*m/z* (Da, tested)	*m/z* (Da, calc)
1	$[Au_2(PPh_3)_2I]^+$	1045.41	1045.4085
2	$[AuCu(PPh_3)_2I_2CH_3CH_2OH]H^+$	1085.94	1085.9688
3	$[Au_9(PPh_3)_8]^{3+}$	1290.96	1290.9501
4	$[Au_6(PPh_3)_6H_2]^{2+}$	1378.57	1378.7640
5	$[Au_3(PPh_3)_3]^+CH_3OH$	1409.81	1409.7980
6	$[Au_8(PPh_3)_7]^{2+}$	1706.12	1705.8654
7	$[Au_8Cu(PPh_3)_8]^{2+}$	1868.78	1868.7811
8	$[Au_8Cu_2(PPh_3)_8I]^{2+}$	1964.01	1964.0063
9	$[Au_{10}Cu_2(PPh_3)_7IH_2]^{2+}$	2030.84	2030.8341
10	$[Au_9(PPh_3)_8(SbF_6)]^{2+}$	2053.35	2053.3666
11	$[Au_9Cu(PPh_3)_8I_2]^{2+}$	2094.17	2094.1689
12	$[Au_6(PPh_3)_5I]^+$	2620.13	2620.1312
13	$[Au_9Cu_2(PPh_3)_7]^+$	3736.03	3735.7894
14	$[Au_8Cu_2(PPh_3)_8I_2]^+$	3871.97	3872.0136
15	$[Au_8Cu_2(PPh_3)_7Cl_2]^+$	4054.89	4054.9172
16	$[Au_8Cu_2(PPh_3)_8I]^+$	3928.02	3928.0127
17	$[Au_8Cu_2(PPh_3)_8I_2HCOO]^+$	4099.95	4099.9346
18	$[Au_9Cu(PPh_3)_8I_2]^+$	4188.28	4188.3378
19	$[Au_8Cu_2(PPh_3)_8IClHCO_2]^+$	4008.45	4008.4829
20	$[Au_{24}Cu_1(PPh_3)_{10}I_7]^{2+}$	4150.95	4150.9655
21	$[Au_{23}Cu_2(PPh_3)_{10}I_7]^{2+}$	4084.32	4084.2552
22	$[Au_{22}Cu_3(PPh_3)_{10}I_7]^{2+}$	4017.56	4017.5449
23	$[Au_{21}Cu_4(PPh_3)_{10}I_7]^{2+}$	3950.90	3950.8346
24	$[Au_{20}Cu_5(PPh_3)_{10}I_7]^{2+}$	3884.21	3884.1243
25	$[Au_{19}Cu_6(PPh_3)_{10}I_7]^{2+}$	3818.50	3817.4140
26	$[Au_{18}Cu_7(PPh_3)_{10}I_7]^{2+}$	3750.68	3750.7037
27	$[Au_{17}Cu_8(PPh_3)_{10}I_7]^{2+}$	3684.05	3683.9934
28	$[Au_{16}Cu_9(PPh_3)_{10}I_7]^{2+}$	3617.31	3617.2831

Supplementary Table 2: List of the average bond lengths and the distances in the $[Au_{25}(PPh_3)_{10}Br_7]^{2+}$ (Au_{25}), and $[Au_{12}Cu_{13}(PPh_3)_{10}I_7]^{2+}$ ($Au_{12}Cu_{13}$) nanoclusters.

Entry	Length (average, Å)	Au_{25}	$Au_{12}Cu_{13}$
1	$Au\text{-}Au_{(core)}$	2.734 (2.707-2.768)	2.736 (2.711-2.768)
2	M(core)−M(Cu/Au)	2.878 (2.851-2.890)	2.717 (2.631-2.777)
3	$Au_{(hemispherical)}$-M(Cu)	2.881 (2.809-2.956)	2.904 (2.776-3.067)
4	Au−P	2.310 (2.297-2.329)	2.297 (2.226-2.342)
5	$M_{(apical\ of\ Cu/Au)}$−X(I/Br)	2.481	2.606 (2.599-2.612)
6	$M_{(equatorial\ of\ Cu/Au)}$−X(I/Br)	2.550 (2.536-2.590)	2.5861 (2.552-2.627)
7	distance of M_5	2.995	2.7251

Supplementary Table 3: Summary of catalytic activity of the selective photo-oxidation of methanol into methyl formate over the various catalysts at 25 °C. X_{CH_3OH}: CH_3OH conversion; S_{MF}: MF-selectivity.

Entry	Catalyst	X_{CH_3OH} (%)	S_{MF} (%)	MF formation rate ($mmol\cdot g^{-1}\cdot h^{-1}$)	Ref.
1	P25	27	56	1.8	4
2	Cu/TiO_2	65	55	4.4	5
3	CuO/CuZnAl	80	60	5.8	6
4	Au/TiO_2	65	75	5.9	4
5	Ag/TiO_2	75	80	7.3	4
6	$Al_2O_3/Au_{12}Cu_{13}/TiO_2$	92	84	10.6	*This work*

Supplementary References

(1) Sheldrick, G. M. SHELXT - integrated space-group and crystal-structure determination. *Acta Crystallogr. A Found. Adv.* **71**, 3-8 (2015).

(2) Sheldrick, G. M. Crystal structure refinement with SHELXL. *Acta Crystallogr. C Struct. Chem.* **71**, 3-8 (2015).

(3) Dolomanov, O. V., Bourhis, L. J., Gildea, R. J., Howard, J. A. K. & Puschmann, H. OLEX2: a complete structure solution, refinement and analysis program. *J. Appl. Crystallogr.* **42**, 339-341 (2009).

(4) Han, C. H. et al. Selective Oxidation of Methanol to Methyl Formate on Catalysts of Au–Ag Alloy Nanoparticles Supported on Titania under UV Irradiation. *Green Chem.* **16**, 3603-3615 (2014).

(5) Liu, J. et al. Methyl Formate Synthesis from Methanol on Titania Supported Copper Catalyst under UV Irradiation at Ambient Condition: Performance and Mechanism. *J. Catal.* **333**, 162-170 (2016).

(6) Liang, X. Y. et al. Performance and Mechanism of CuO/CuZnAl Hydrotalcites-ZnO for Photocatalytic Selective Oxidation of Gaseous Methanol to Methyl Formate at Ambient Temperature. *J. Catal*. **339**, 68-76 (2016).
