# Supplementary material for: Atomically precise copper dopants in metal clusters boost up stability, fluorescence, and photocatalytic activity
Source: Commun Chem. 2023 Feb 8;6:24. doi: 10.1038/s42004-023-00817-5 (PMC9908894; doi:10.1038/s42004-023-00817-5)
Supplement: Supplementary file 3 — Description of Additional Supplementary Files [file 42004_2023_817_MOESM3_ESM.pdf]

Description of Additional Supplementary Files

File name: Supplementary Data 1

Description: Cif for [Au12Cu13(Ph3P)10I7](SbF6)2

File name: Supplementary Data 2

Description: Cif for [Au25(Ph3P)10Br7](SbF6)2
